# Changes in bone density and microarchitecture in adolescents undergoing a first kidney transplantation: a prospective study

**DOI:** 10.1007/s00431-024-05777-z

**Published:** 2024-10-09

**Authors:** Aurélie De Mul, Anne-Laure Sellier Leclerc, Tiphanie Ginhoux, Charlène Levi, Cyrille Confavreux, Manon Aurelle, Aurélie Portefaix, Justine Bacchetta

**Affiliations:** 1 Centre de Référence Des Maladies Rénales Rares, Centre de Référence Des Maladies Rares du Calcium Et du Phosphore, Filières Maladies Rares ORKiD, OSCAR Et ERK-Net, Hôpital Femme Mère Enfant, 69677 Bron, France; 2 Clinical Investigation Center (CIC 1407), Hospices Civils de Lyon, 69677 Bron, France; 3 Service de Transplantation, Néphrologie Et Immunologie Cliniques, Hôpital Edouard Herriot, Hospices Civils de Lyon, 69003 Lyon, France; 4 Service de Rhumatologie, Hôpital Lyon Sud, Hospices Civils de Lyon, 69310 Lyon, Pierre-Bénite France; 5 Faculté de Médecine Lyon Est, Université Claude Bernard Lyon I, 69003 Lyon, France; 6 INSERM UMR 1033 LYOS, 69372 Lyon, France; 7 Laboratoire de Biométrie Et Biologie Evolutive, CNRS, UMR 5558, Université Claude Bernard Lyon I, Villeurbanne, 69622 Lyon, France

**Keywords:** Children, CKD-MBD, HR-pQCT, Kidney transplantation

## Abstract

**Purpose:**

Mineral bone disorder associated with chronic kidney disease (CKD-MBD) frequently persists after kidney transplantation (KTx), being due to pre-existing CKD-MBD, immunosuppressive therapies, and post-KTx hypophosphatemia. This study aimed to evaluate bone biomarkers and microarchitecture using high resolution peripheral quantitative computed tomography (HR-pQCT) at the time of KTx and 6 months thereafter and to compare these results with those of matched healthy controls (HC).

**Methods:**

This study presented the single-center subgroup of patients aged between 10 and 18 years included in the prospective “Bone Microarchitecture in the Transplant Patient” study (TRANSOS-NCT02729142). Patients undergoing a first KTx were matched (1:2) with HC from the “Vitamin D, Bones, Nutritional and Cardiovascular Status” cohort (VITADOS) on sex, pubertal stage, and age.

**Results:**

At a median (interquartile range, IQR) age of 15 [13; 16] years, 19 patients (6 girls, 7 pre-emptive KTx, 7 steroid-sparing immunosuppressive strategies) underwent a first KTx, with a median [IQR] parathyroid hormone level of 1.9 [1.4; 2.9] the upper limit of normal (ULN). Higher total and trabecular bone densities, along with superior trabecular microarchitecture, were observed at KTx compared to HC. Six months post-KTx, patients had significantly impaired trabecular parameters at the radius, while results were not significantly different at the weight-bearing tibia, neither cortical parameters at both sites. Six months post-KTx, 6 (32%) patients still present with metabolic acidosis, 10 (53%) persistent hyperparathyroidism (always < 2 ULN), and 5 (26%) elevated FGF23 levels; 11 (58%) received phosphate supplementation.

*Conclusions*: Bone density and microarchitecture at the time of KTx were superior compared to HC, but radial trabecular bone microarchitecture impairment observed 6 months post-KTx may reflect subtle albeit present post-KTx CKD-MBD.

**Table:** 

**What is Known?** • *Mineral bone disorder associated with chronic kidney disease (CKD-MBD) frequently persists after kidney transplantation (KTx) and is associated with morbidity. However, biochemical parameters and dual X-ray absorptiometry (DXA) are poor predictors of the underlying bone disease.*
**What is new?** • * The present study on 19 adolescent KTx recipients with adequate CKD-MBD control at the time of KTx reveals no significant bone disease compared to matched healthy controls. Microarchitecture impairment observes 6 months post-KTx may reflect subtle, albeit present, post-KTx CKD-MBD.*

**Supplementary Information:**

The online version contains supplementary material available at 10.1007/s00431-024-05777-z.

## Introduction

During childhood, common symptoms of mineral and bone disorder associated with chronic kidney disease (CKD-MBD) include growth retardation, bone pain, fractures, skeletal deformities, osteopenia, osteonecrosis, and vascular calcifications. Frequently persisting after kidney transplantation (KTx), CKD-MBD is associated with significant morbidity [1–5]. Up to 40 percent of pediatric KTx recipients describe bone-related symptoms [1, 2]. The rate of vertebral and peripheral fractures is high, with an incidence of all fractures sixfold higher and vertebral fractures 160-fold higher in the pediatric KTx recipients compared with control populations [3]. In addition, around half of pediatric KTx recipients display persistent short stature [4].

Risk factors include pre-existing CKD-MBD and renal osteodystrophy, immunosuppressive therapies including not only corticosteroids but also calcineurin inhibitors and mTor inhibitors, reduced graft function, metabolic acidosis, malnutrition, hypogonadism, reduced physical activity, as well as disruption of the phosphocalcic axis (i.e., parathyroid hormone (PTH), 25-hydroxyvitamin D, and fibroblast growth factor 23 (FGF23) axis), leading to persistently elevated FGF23 and PTH levels for weeks/months post-KTx that further induce hypophosphatemia and subsequent mineralization defects [6–8].

Even if it is rarely performed in routine, bone biopsy is the gold standard technique to assess lesions of bone turnover and mineralization: Sanchez et al. describe in 47 pediatric KTx recipient’s persistent renal osteodystrophy in 30% of the patients, with adynamic bone disease in 10% and high turnover lesions in 23% [9]. Unfortunately, biochemical parameters are poor predictors of the underlying renal osteodystrophy [10]; furthermore, the most widely used two-dimensional imaging technique to assess skeletal mineral content and structure, namely dual X-ray absorptiometry (DXA), does not correlate with fracture risk nor with the underlying bone disease and is usually normal in pediatric KTx recipients after correction for growth retardation [11, 12]; DXA is not recommended in daily practice for the follow-up of pediatric CKD-MBD [10]. To overcome these drawbacks, HR-pQCT offers a more informative tridimensional imaging technique by providing both volumetric and microarchitectural bone evaluations [13]. This research tool has proved its value in pediatric CKD-MBD [14–17], but has rarely been performed in pediatric KTx recipients [18–21].

The aims of this prospective study were to determine the evolution of HR-pQCT parameters and bone biomarkers in adolescents receiving a first KTx between KTx and 6 months post-KTx, but also to compare their bone parameters to matched healthy controls (HC).

## Patients and methods

### Study design and population

Pediatric patients who had undergone a first KTx and were aged between 10 and 18 years at the time of inclusion in the single-center prospective “Bone Microarchitecture in the Transplant Patient Study” (TRANSOS-NCT02729142- March 25, 2016) were included in the present study. The overall aim of the TRANSOS study was to evaluate the evolution of bone biomarkers, DXA, and HR-pQCT in adolescents and adults receiving a first KTx or a combined pancreas/kidney transplantation in the pediatric nephrology and the adult transplantation units of the Hospices Civils de Lyon, as well as the adult transplantation unit of the Hospital Universitaire de Saint Etienne.

Bone status was evaluated at the time of KTx and 6 months post-KTx by performing DXA and HR-pQCT. Given that transplantations primarily involve organs from deceased donors and are not scheduled events, the initial bone imaging evaluations (referred to as “at KTx”) could be conducted until the end of the fourth week after transplantation. Clinical and biochemical data were collected at the exact time of KTx (just before the surgical procedure), 1-month post-KTx and 6 months post-KTx. Each patient was matched on age (± one year), sex, and pubertal stage (Tanner stage) to two HC from the local pediatric “Vitamin D, Bones, Nutritional and Cardiovascular Status” cohort (VITADOS-NCT01832623-April 16, 2013) [22]. If more than two subjects were available, the selected control subjects were the ones closest in height to the patient. A local independent ethics committee (CPP Lyon Sud-Est II) and the French national medicines agency (*Agence nationale de sécurité du médicament et des produits de santé*, ANSM) approved the study, respectively in February and March 2016. A written informed consent from parents/legal guardians were required. The research was conducted in accordance with the Declaration of Helsinki.

### Patient characteristics

The following clinical data were collected: height and body weight in standard deviation units according to French pediatric growth charts, and pubertal stage [23]. Causes of kidney failure, dialysis and transplantation characteristics, presence or absence of any bone-related symptom (history of fracture, bone deformation, and/or bone pain), hospitalization after KTx, immunosuppressive therapy, bicarbonate, and phosphate supplementation were collected.

### Physical activity

A self-administered questionnaire was used to evaluate physical activity over a 7-day period including exercise and sports performed at school, at home or in a club, but also activities from daily life such as house duties, way to school, and artistic activities [24]. All forms of physical activity associated with a given metabolic equivalent (MET) according to the intensity of activity as determined previously [25], and, for the most common, MET were validated using the reference standard, namely double-labeled water [24]. One MET corresponds to 1 kcal/kg per hour or 4.184 kJ/kg per hour. The estimated physical activity results were reported as MET per day. KTx recipients were prohibited from engaging in intense physical activity during the first month following transplantation. Furthermore, the patients were proposed adapted physical activity follow-up at the day-care hospital.

### Biomarkers

Serum calcium, phosphate, magnesium, bicarbonate (metabolic acidosis usually defined in this population as bicarbonate < 22 mmol/L on a non-hemolyzed venous sample), standardized creatinine determined by isotope-dilution mass spectrometry, intact third generation PTH (chemiluminescence assay, Liaison XL DiaSorin analyzers, DiaSorin reagent; normal values 5.5–38.4 pg/ml), 25-hydroxyvitamin D (electrochemiluminescence assay, Cobas e analyzers, Elecsys Roche reagent; target range 75–120 nmol/l), 1,25-dihydroxyvitamin D (chemiluminescence assay, Liaison XL DiaSorin analyzers, DiaSorin reagent; normal values 69–200 pmol/l), and total alkaline phosphatase (ALP) were measured on fasting blood samples in the morning using routine methods in the same laboratory. C-terminal second generation FGF23 (Enzyme-Linked Immunosorbent Assay, Immutopics), total serum osteocalcin (chemiluminescence assay, Liaison XL DiaSorin analyzers, DiaSorin reagent), and serum β-crosslaps (electrochemiluminescence assay, Cobas e analyzers, Elecsys Roche reagents) were assessed; local reference values have been previously published in adolescents according to pubertal stage and sex [22].

### Bone-imaging techniques

Two bone-imaging techniques were used for bone evaluation, namely DXA and HR-pQCT. DXA Z-scores of the spine and total body, were calculated according to age, pubertal stage, and sex reference charts. Lean mass (kg) and fat mass (kg) were calculated from the whole body DXA scan excluding the skull. As previously described [16], bone density as well as cortical and trabecular microstructure were assessed at the non-dominant radius and tibia by performing HR-pQCT (XtremeCT; Scanco Medical AG,) with the following settings: effective energy of 60 kVp, X-ray tube current of 900 μA, and matrix size of 1536 × 1536. A stack of 110 parallel computed tomography slices was acquired leading to a three-dimensional representation of 9 mm in the axial direction (voxel size 82 μm^3^). The total scan time was 2.8 min with a low radiation exposure of approximately 3 μSv. This technique has excellent precision for both structural parameters (1.5–4.4%) and volumetric bone mineral density (0.7–1.5%) [13]. A trained operator generates semi-automatic contours around the periosteal surface and the entire volume of interest is automatically separated into a cortical and trabecular region. The variables include volumetric bone mineral density (vBMD; mg/cm^3^) for total (Tt.vBMD), trabecular (Tb.vBMD), and cortical (Ct.vBMD) compartments: cortical thickness (Ct.Th; mm), trabecular number (Tb.N; mm^−1^), trabecular thickness (Tb.Th; mm), trabecular separation (Tb.Sp; mm), cortical area (Ct.Ar; mm^2^), and total area (T.Ar; mm^2^).

### Statistical analyses

Results are presented as median [interquartile range, IQR] for quantitative data and number (percentage) for qualitative data. Non-parametric tests were used for comparisons: the Mann–Whitney test to compare HC and patients at KTx; the Wilcoxon test for paired groups to compare patients at KTx and six months post-KTx; and the Friedman test and its post hoc test to compare patients at KTx, one-month post-KTx, and six months post-KTx. To assess the correlations between physical activity using MET/day and bone parameters, Spearman bivariate analyses were performed. Exploratory subgroup analyses were conducted 6 months post-KTx to compare the percentage change in HR-pQCT values between measurements taken at 6 months post-KTx and those obtained at KTx using Wilcoxon test. Statistical significance was adjusted using Bonferroni correction to account for multiple group comparisons. Statistical analyses were performed using the SPSS software version 17.0 (SPSS, Inc.).

## Results

### Patients

A total of 19 pediatric KTx recipients (six girls) were included; these had a median [IQR] age of 15 [13; 16] years and were matched to 38 HC (12 girls) the median [IQR] age of whom was 15 [13; 16] years (*p* = 0.52). The median [IQR] pubertal stage at transplantation was 4 [2; 4], which did not significantly differ from HC (4 [2; 4], *p* = 0.7). None of the patients had a genetic disease with specific bone involvement. Seven patients underwent preemptive KTx and seven received steroid-sparing immunosuppression. No patient developed fractures, presented bone pain, or skeletal deformities during follow-up, but three had a history of high kinetic energy trauma fracture. The median [IQR] duration of hospitalization following KTx was 14 [12; 16] days. During the 6-month follow-up period, three patients were hospitalized due to a rise in creatinine requiring kidney biopsy. Among them, one patient experienced acute cellular rejection and was treated with a 5-day course of intravenous corticosteroid pulse followed by a tapering regimen of oral corticosteroids. For the other two patients, acute kidney injury was attributed to acute tubular injury resulting from dehydration. Four patients developed infectious illness requiring hospitalization (bronchitis, pyelonephritis, gastroenteritis, and febrile neutropenia). No other hospitalization occurred. Clinical data of patients are presented in Table 1. Weight standard deviation score (SDS) improved from KTx to 6 months post-KTx without reaching significance. Anthropometric data for patients and HC is presented in Table 2. There was a trend towards a lower median [IQR] estimated physical activity at KTx (12.7 MET/day [11.0; 13.9]) compared to matched HC (13.5 MET/day [10.3; 18.6]), *p* = 0.24). There was also a trend towards a lower median [IQR] estimated physical activity six months post-KTx (12 MET/day [9.3–13.6]) compared to that at KTx (12.7 MET/day [11.0; 13.9], *p* = 0.27).

**Table 1:** Patient characteristics

Total population, *N* (%)	19 (100)
Age (years), median [IQR]	15 [13;16]
Girls, *N* (%)	6 (32)
Underlying renal diagnosis	
Hereditary nephropathy, *N* (%)	8 (42)
CAKUT, *N* (%)	6 (32)
Glomerulopathy, *N* (%)	2 (11)
Undetermined nephropathy, *N* (%)	2 (11)
Polycystic disease, *N* (%)	1 (5)
Pre-emptive transplantation, *N* (%)	7 (37)
Living donor, *N* (%)	4 (21)
Dialysis	
Hemodialysis, *N* (%)	1 (5)
Peritoneal dialysis, *N* (%)	11 (58)
Duration of dialysis (months), median [IQR]	6.1 [3.1; 12.0]
CKD-MBD manifestations	
Fracture history, *N* (%)	3 (16)
Post-KTx follow-up	
Bone pain, *N*	0
Skeletal deformities, *N*	0
Fracture, *N*	0
Immunosuppressive drug scheme at transplantation	
Basiliximab, *N* (%)	19 (100)
Tacrolimus, *N* (%)	18 (95)
Mycophenolate Mofetil, *N* (%)	19 (100)
Eculizumab, *N* (%)	1 (5)
Corticosteroids, *N* (%)	19 (100)
Immunosuppressive drug scheme at 6 months	
Tacrolimus, *N* (%)	19 (100)
Mycophenolate mofetil (%)	16 (84)
Azathioprine, N (%)	2 (11)
Everolimus, *N* (%)	1 (5)
Eculizumab, *N* (%)	1 (5)
No corticosteroids, *N* (%)	7 (37)

*CAKUT* congenital anomalies kidney and urinary tract, *IQR* interquartile range, *KTx* kidney transplantation

**Table 2:** Biometry

	At KTx		6 months post-KTx		*Healthy controls*	
Height SDS	− 0.6*	[− 1.2; − 0.14]	− 0.7	[− 1.3; 0.0]	*0.3*	*[*− *0.5; 1.2]*
Weight SDS	− 1.0*	[− 2.0; 0.1]	− 0.5	[− 1.7; 0.6]	*0.7*	*[*− *0.4; 1.4]*
BMI Z-scores for girls	− 0.8	[− 1.4; − 0.1]	− 0.2	[− 0.8; 0.7]	− *0.2*	*[*− *1.4; 0.0]*
BMI Z-scores for boys	− 0.9	[− 1.1; − 0.4]	− 0.2	[− 0.8; 0.7]	*0.4*	*[*− *1.1; 0.2]*

Results expressed as median [interquartile range]. *SDS* standard deviation score, *BMI* body mass index (kg/m^2^)

*p < 0.025 when comparing HC and patients at KTx

^#^*p* < 0.025 when comparing patients at KTx and 6 months post-KTx

### Biomarkers

The median [IQR] PTH at KTx was 74 [53; 113] pg/ml, corresponding to a median [IQR] 1.9 [1.4; 2.9] the upper limit of normal (ULN). SDS for median [IQR] phosphate at the time of kidney transplantation was 0.5 [− 0.35; 1.86]. Phosphate, PTH, FGF23, β-crosslaps, and osteocalcin decreased between KTx and six months post-KTx, and were higher compared to HC. Bicarbonate values and 1.25 dihydroxy vitamin D were higher in HC compared to patients at KTx. In contrast, no difference was observed for calcium and ALP. Six months post-KTx, 6 (32%) patients still present with metabolic acidosis, 10 (53%) persistent hyperparathyroidism (but always < 2 ULN), and 5 (26%) elevated FGF23 levels. In terms of management,6 months post-KTx, 11 patients (58%) received phosphate supplementation, and 10 (53%) alkali supplementation (i.e., sodium bicarbonate). Despite native vitamin D supplementation, only one patient had 25-hydroxyvitamin D within the target range at six months post-KTx. No seasonal differences for 25-hydroxyvitamin D were found. The median [IQR] 25-hydroxyvitamin D at KTx during winter was 78 [66; 90] nmol/l, and median [IQR] 25-hydroxyvitamin D at KTx during summer was 68 [64; 76] nmol/l (*p* = 0.29). The median [IQR] 25-hydroxyvitamin D at 6 months post-KTx during winter was 43 [37; 54] nmol/l, and median [IQR] 25-hydroxyvitamin D at 6 months post-KTx during summer was 54 [46; 64] nmol/l (*p* = 0.36). Biochemical parameters for patients and HC are presented in Table 3.

**Table 3:** Laboratory parameters

	At KTx		1 month post-KTx		6 months post-KTx		*Healthy controls*	
Creatinine (µmol/l)			96	[79; 108]	83	[69; 99]	*61*	*[53; 72]*
Bicarbonate (mmol/l)	22*	[19; 23]	21	[20; 23]	22	[20; 23]	*27*	*[25; 28.2]*
Calcium (mmol/l)	2.41	[2.29; 2.53]	2.45	[2.40; 2.55]	2.45	[2.41; 2.53]	*2.41*	*[2.36; 2.53]*
Phosphate (mmol/l)	1.56*#§¤	[1.34; 1.83]	1.09	[0.97; 1.35]	1.25	[1.11; 1.42]	*1.31*	*[1.11; 1.45]*
Phosphate SDS	0.50*#§¤	[− 0.35; 1.86]	− 1.80	[− 2.43; − 0.62]	− 1.30	[− 1.65; 0.15]	− *0.95*	*[*− *1.47;* − *0.31]*
ALP (UI/l)^a^	156	[101; 268]	136	[110; 185]	168	[107; 202]	*150*	*[90; 210]*
PTH (pg/ml)	74*#	[53; 113]	29	[21; 40]	39	[27; 41]	*17*	*[14; 23]*
PTH ULN	1.9*#	[1.4; 2.9]	0.7	[0.5; 1.0]	1.0	[0.7; 1.1]	*0.4*	*[0.4; 0.6]*
25-OH vitamin D (nmol/L)	68*¤	[58; 78]	65	[55; 77]	47	[38; 57]	*63*	*[50; 86]*
1,25 OH vitamin D (pmol/l)	64*#¤@	[23; 91]	65	[22; 132]	134	[98; 164]	*126*	*[92; 152]*
FGF23 (RU/ml)	1002*#§¤	[347; 1642]	108	[85; 174]	118	[76; 165]	*50*	*[40; 62]*
β-Crosslaps (pg/ml)^b^	3366*	[2715; 4339]	2223	[1636; 2960]	2769	[1790; 3276]	*1421*	*[971; 1734]*
Osteocalcin (μg/L)	355 *#§	[290; 390]	66	[43; 124]	93	[65; 137]	*59*	*[38; 81]*

Results expressed as median [interquartile range] *ALP* alkaline phosphatase, *FGF23* fibroblast growth factor 23, *PTH* parathyroid hormone, *KTx* kidney transplantation, *SDS* standard deviation score, *ULN* upper limit of normal

*p < 0.0125 when comparing HC and patients at KTx

^#^*p* < 0.0125 when comparing patients at KTx, 1 month post-KTx and 6 months post-KTx

^§^*p* < 0.0125 when comparing patients at KTx and 1 month post-KTx; ¤ p < 0.0125 when comparing patients at KTx and 6 months post-KTx; @*p* < 0.0125 when comparing patients at 1 month and 6 after KTx

^a^Five missing data at KTx, 4 missing data 1 months post-KTx and 3 missing data 6 months post-KTx; ^b^two missing data

### Bone imaging

#### DXA

There was a trend towards a higher median [IQR] whole-body bone mineral content Z-scores among HC (0.1 [− 0.5; 0.9]) compared to patients at the time of KTx (− 0.4 [− 1.5; 0.2]), *p* = 0.04). Patients at the time of KTx had significantly higher median [IQR] whole-body bone mineral content Z-scores (− 0.4 [− 1.5; 0.2]) compared to 6 months post-KTx (− 0.6 [− 1.8; 0.1], *p* = 0.002). The median [IQR] spine DXA Z-scores were not significantly different at KTx.

(− 0.8 [− 1.2; 0.2]) compared to 6 months post-KTx (− 0.8 [− 1.3; 0.0], *p* = 0.05) and to HC (− 0.35 [− 0.7; 0.4], *p* = 0.12). The median [IQR] lean body mass (kg) was significantly lower at the time KTx (37 [34; 40] kg) compared to 6 months post-KTx (40 [36; 43] kg, *p* < 0.001) and to HC (44 [36; 50] kg, *p* = 0.009).

#### HR-pQCT

Total and trabecular densities at the radius were significantly higher at the time of KTx compared both to HC and to 6 months post-KTx. Similarly, trabecular micro-architecture was significantly better at the time of KTx compared to HC and to 6 months post-KTx. No significant difference was observed on cortical parameters at the radius. HR-pQCT results at the radius are presented in Table 4. In contrast, at the weight-bearing tibia, no significant difference was observed between HC and patients, or between the time of KTx and six months post-KTx. HR-pQCT results at the tibia are presented in Table 5. No correlations were observed between estimated physical activity measured in MET/day and bone parameters.

**Table 4:** Radial HR-pQCT

	At KTx		6 months post-KTx		*Healthy controls*	
Density						
Tb.vBMD (mg/cm^3^)	238^*#^	[210; 265]	217	[190; 246]	*182*	*[164; 213]*
Tt.vBMD (mg/cm^3^)	322^*#^	[300; 354]	306	[284; 331]	*285*	*[250; 315]*
Ct.vBMD (mg/cm^3^)	721	[639; 790]	717	[641; 768]	*712*	*[618; 780]*
Trabecular structure						
Tb.N (mm^−1^)	2.10	[1.93; 2.21]	2.04	[1.94; 2.14]	*2.00*	*[1.80; 2.12]*
Tb.Th (mm)	0.09^*#^	[0.09; 0.10]	0.09	[0.08; 0.09]	*0.08*	*[0.07; 0.09]*
Tb.Sp (mm)	0.38^*#^	[0.35; 0.43]	0.40	[0.37; 0.44]	*0.44*	*[0.39; 0.47]*
Geometry						
Ct.Ar (mm^2^)	33	[18; 46]	30	[22; 44]	*31*	*[19; 51]*
Tt.Ar (mm^2^)	183	[160; 219]	184	[163; 219]	*216*	*[172; 248]*
Ct.Th (mm)	0.55	[0.33; 0.69]	0.53	[0.37; 0.68]	*0.54*	*[0.32; 0.78]*

Results expressed as median [interquartile range]. *vBMD* volumetric bone mineral density for total (Tt.vBMD), trabecular (Tb.vBMD), and cortical (Ct.vBMD) compartment, *Tb.N* trabecular number, *Tb.Th* thickness, *Tb.Sp* separation, *Ct.Ar* cortical area, *Tt.Ar* total area, *Ct.Th* cortical thickness, *KTx* kidney transplantation

*p < 0.025 when comparing HC and patients at KTx

^#^*p* < 0.025 when comparing patients at KTx and 6 months post-KTx

**Table 5:** Tibial HR-pQCT

	At KTx		6 months post-KTx		*Healthy controls*	
Density						
Tb.vBMD (mg/cm^3^)	203	[188; 224]	204	[173; 219]	*190*	*[180; 207]*
Tt.vBMD (mg/cm^3^)	288	[264; 320]	287	[264; 321]	*271*	*[252; 335]*
Ct.vBMD (mg/cm^3^)	797	[729; 876]	791	[738; 876]	*781*	*[715; 869]*
Trabecular structure						
Tb.N (mm^−1^)	1.72	[1.54; 2.01]	1.73	[1.54; 1.93]	*1.81*	*[1.68; 1.94]*
Tb.Th (mm)	0.10	[0.09; 0.11]	0.10	[0.09; 0.11]	*0.09*	*[0.08; 0.09]*
Tb.Sp (mm)	0.47	[0.42; 0.54]	0.47	[0.43; 0.55]	*0.46*	*[0.43; 0.51]*
Geometry						
Ct.Ar (mm^2^)	83	[51; 93]	81	[63; 98]	*104*	*[73; 123]*
Tt.Ar (mm^2^)	510	[396; 613]	512	[380; 617]	*581*	*[473; 653]*
Ct.Th (mm)	0.84	[0.56; 1.02]	0.85	[0.69; 1.03]	*1.02*	*[0.77; 1.20]*

Results expressed as median [interquartile range, IQR]. *vBMD* volumetric bone mineral density for total (Tt.vBMD), trabecular (Tb.vBMD), and cortical (Ct.vBMD)compartment, *Tb.N* trabecular number, *Tb.Th* thickness, *Tb.Sp* separation, *Ct.Ar* cortical area, *Tt.Ar* total area, *Ct.Th* cortical thickness, *KTx* kidney transplantation

*p < 0.025 when comparing HC and patients at KTx

^#^*p* < 0.025 when comparing patients at KTx and 6 months post-KTx

Exploratory subgroup analyses were performed: No significant difference was observed for HR-pQCT parameters between patients with or without steroid-sparing immunosuppression, between patients with or without alkali supplementation at 6 months post-KTx, between patients with persistent hyperparathyroidism or not at 6 months post-KTx, or between patients with or without preemptive transplantation (Supplementary Material Table S1).

## Discussion

Using HR-pQCT, an accurate and non-invasive technique with low radiation exposure for bone evaluation, bone quantity, and quality in 19 adolescent KTx recipients, who had tightly controlled bone biomarkers at the time of KTx (median PTH levels at 1.9 ULN and phosphate levels at 0.5 SDS), were superior compared to matched healthy controls with dietary calcium intake deficiencies. A slight degradation of trabecular microarchitecture 6 months post-KTx at the radius was observed.

Major changes in serum mineral metabolism occur immediately post-KTx, as thoughtfully described in this cohort during the first 6 months post-KTx. Notably, PTH levels usually decrease, but persistent secondary hyperparathyroidism is described in 10 to 60% of patients 12 months post-KTx [27–29] with hyperparathyroidism being an independent risk factor for allograft dysfunction in pediatric KTx [30]. In this study, 6 months post-KTx, more than half of patients still had elevated PTH levels (but always < 2 ULN). Hyperparathyroidism and persistent elevated circulating FGF3 levels promote renal phosphate wasting and low calcitriol levels [31]. According to data from the Cooperative European Pediatric Renal Transplant Initiative (CERTAIN) registry, more than one-in-ten patients had hypophosphatemia 12 months after KTx, correlated with FGF23 levels [30]. Herein, nearly 60% of the patients received phosphate supplementation 6 months post-KTx, and a quarter of patients had elevated FGF23 levels. The value of phosphate supplementation in this setting remains debated, but one should not forget that hypophosphatemia is a main driver of mineralization defects. On the other hand, giving too much phosphate on a renal transplant with normal or near-normal renal function may also induce secondary hyperparathyroidism, thus justifying a close and regular control of CKD-MBD parameters post-pediatric KTx. Of note, even though the value of cinacalcet after pediatric KTx has been studied [32], none of the patients included herein received cinacalcet during the study period. Another laboratory parameter of importance in the monitoring of CKD-MBD is 25-hydroxyvitamin D as this is also a natural inhibitor of the mTor pathway [33] and that native vitamin D supplementation can prevent the onset of hyperparathyroidism in pediatric CKD [34]. Herein, only one patient had vitamin D within the target range 6 months after KTx, despite native vitamin D supplementation. This finding is consistent with Shroff et al. who reported that 8/106 (7.5%) pediatric KTx recipients were within the target range [35]. This highlights that CKD-MBD is closely controlled during dialysis but may receive less attention post-KTx due to other comorbidities and KTx-specific follow-up needs. Consequently, a close follow-up of 25-hydroxyvitamin D levels and adequate correction of vitamin D deficiency is necessary [36]. In addition, metabolic acidosis, which is associated with altered growth hormone axis, protein degradation,and glucocorticoid production [37], was found in nearly a third of patients herein. Although these various laboratory parameters are poorly correlated with bone histomorphometry, they should be regularly monitored after transplantation in accordance with KDIGO guidelines, with particular attention to trends [38, 39]. Other biomarkers, which are not routinely monitored (β-crosslaps and osteocalcin), were found here to be higher in patients compared to HC, and this can be attributed to their accumulation in cases of kidney failure and the time for these biomarkers to return to levels comparable to those seen in HC.

In the present study, bone evaluation was conducted using two different imaging techniques. DXA, a traditional two-dimensional imaging method not recommended for routine use in the follow-up of pediatric CKD-MBD [10], revealed mainly a significant increase in lean body mass, likely reflecting re-nutrition. In contrast, few pediatric studies exploring the post-KTx period have utilized HR-pQCT [18–21], a more accurate, non-invasive technique with low radiation exposure. HR-pQCT provides distinct compartmental (cortical and trabecular bone) evaluation and characterization of bone microarchitecture, yielding unique insights into bone status compared to DXA. In a cross-sectional study of 55 adolescent KTx recipients, Ruth et al. (2004) reported a decrease in cortical thickness at a mean 4.9 years after transplantation using HR-pQCT [21]. In a longitudinal study of 14 adolescent KTx recipients, Terpstra et al. (2012) reported a significantly lower trabecular vBMD Z-scores 12 months after KTx in association with greater glucocorticoid exposure; in addition, the authors report cortical bone loss at transplantation associated with hyperparathyroidism severity and a significant increase of cortical vBMD Z-scores 12 months after KTx, associated with lower glucocorticoid exposure and lower PTH levels [19]. However, the study by Terpstra et al. is characterized by a high median PTH level (3.6 ULN) at KTx and a high fracture rate in the first 6 months after KTx (10%); it is, therefore, not comparable with the one reported herein. Indeed, the superior bone density and microarchitecture observed at KTx compared to the control HC can be attributed to the very rigorous monitoring and control of mineral biomarkers prior to transplantation and during dialysis. This is exemplified by the tightly regulated median PTH levels (1.9 ULN) at the time of transplantation, as well as well-controlled phosphate levels (0.5 SDS). Additionally, no fractures were observed during the six-month follow-up. Moreover, the strict nutritional control during the pre-dialysis and dialysis phases, with a strong focus on calcium intake in accordance with international guidelines [40, 41], likely contributed to a higher dietary calcium intake in patients at KTx compared to JC. In contrast, the VITADOS cohort, from which the control group was drawn, exhibited significant dietary calcium intake deficiencies, with a median intake of 812 mg (range: 132–1382 mg). These results are comparable with those of a recent study using the same HR-pQCT technique in 32 adolescents from Lyon who were part of the 4C study, although the inclusion criteria were different (CKD patients with a median GFR of 33 mL/min per 1.73 m^2^). In that study, bone compartmental densities and microarchitecture in children with moderate CKD and tightly controlled mineral biomarkers (median PTH 1.2 ULN) were not found to be significantly worse compared to healthy controls [14]. However, it is of note that a slight degradation of trabecular microarchitecture 6 months post-KTx at the radius was observed herein. Such impairment may be partly explained by a less rigorous control of mineral metabolism post-KTX but also by the combination of bone risk factors that is maximal in the early post-KTx period, such as the use of corticosteroids and calcineurin inhibitors, and the presence (even if supplemented) of hypophosphatemia and metabolic acidosis.

The limitations of the present study include a small sample size, which limits the statistical power to demonstrate a potential impact of corticosteroids, and the possibility to adjust for important factors like physical activity. Other limitations are a short follow-up period after KTx and the use of a self-administered questionnaire for assessing physical activity that was chosen for its practicality over more complex methods such as double-labeled water techniques or pedometer. It would be valuable to conduct a supplementary study involving HR-pQCT scans 12 months or more after KTx.

## Conclusion

Adolescent transplant recipients with well-controlled CKD-MBD at the time of KTx demonstrated superior bone density and microarchitecture compared to matched healthy controls, as evaluated using HR-pQCT, a comprehensive and rarely performed three-dimensional bone assessment technique. However, observed impairment in radial trabecular bone microarchitecture 6 months post-KTx may reflect a combination of bone risk factors that are most pronounced in the early post-KTx period.

## Supplementary Information

Below is the link to the electronic supplementary material.

Supplementary file1 (DOCX 16.3 KB)

## Data Availability

No datasets were generated or analysed during the current study.

## Abbreviations

ALP: Alkaline phosphatase
BMI: Body mass index
CKD: Chronic kidney disease
CKD-MBD: Mineral bone disorder associated with chronic kidney disease
Ct.Ar: Cortical area
Ct.vBMD: Cortical volumetric bone mineral density
Ct.Th: Cortical thickness
DXA: Dual X-ray absorptiometry
FGF23: Fibroblast growth factor 23
HC: Healthy controls
IQR: Interquartile range
HR-pQCT: High resolution peripheral quantitative computed tomography
KTx: Kidney transplantation
MET: Metabolic equivalent
PTH: Parathyroid hormone
SDS: Standard deviation score
Tt.Ar: Total area
Tb.N: Trabecular number
Tb.Sp: Trabecular separation
Tb.Th: Trabecular thickness
Tb.vBMD: Trabecular volumetric bone mineral density
TRANSOS: Bone Microarchitecture in the Transplant Patient Study
Tt.vBMD: Total volumetric bone mineral density
ULN: Upper limit of normal
vBMD: Volumetric bone mineral density
VITADOS: Vitamin D, Bones, Nutritional and Cardiovascular Status cohort
