# Supplementary material for: Changes in bone density and microarchitecture in adolescents undergoing a first kidney transplantation: a prospective study
Source: Eur J Pediatr. 2024 Oct 9;183(12):5303–12. doi: 10.1007/s00431-024-05777-z (PMC11527940; doi:10.1007/s00431-024-05777-z)
Supplement: Supplementary file 1 — Supplementary file1 (DOCX 16.3 KB) [file 431_2024_5777_MOESM1_ESM.docx]

| **Supplementary Material-Table 1** | | | | |
| --- | --- | --- | --- | --- |
| Radial HR-pQCT | **Corticosteroids** (n=12) | | **No corticosteroids** (n=7) | |
| **Density** | | | | |
| ∆Tb.vBMD (mg/cm^3^) | -10.1 | [-15.6 ; -3.7] | -7.3 | [-13.1 ; -3.9] |
| ∆Tt.vBMD (mg/cm^3^) | -5.0 | [-7.6 ; -2.2] | -6.0 | [-9.0 ; -1.3] |
| ∆Ct.vBMD (mg/cm^3^) | 1.2 | [-3.0 ; 4.3] | -2.4 | [-3.3 ; 2.0] |
| **Trabecular structure** | | | | |
| ∆Tb.N (mm^−1^) | -0.4 | [-3.7 ; 1.7] | -2.3 | [-5.0 ; 0.1] |
| ∆Tb.Th (mm) | -9.8 | [-16.2 ; -1.3] | -4.6 | [-9.2 ; 0.0] |
| ∆Tb.Sp (mm) | 2.1 | [0.4 ; 8.2] | 3.2 | [0.0 ; 7.8] |

| Radial HR-pQCT | **Metabolic acidosis** (n=9) | | **No metabolic acidosis** (n=10) | |
| --- | --- | --- | --- | --- |
| **Density** | | | | |
| ∆Tb.vBMD (mg/cm^3^) | -13.3 | [-15.4 ; -1.0] | -5.6 | [-12.0 ; -4.1] |
| ∆Tt.vBMD (mg/cm^3^) | -6.6 | [-8.7 ; -1.9] | -4.5 | [-9.0 ; -1.0] |
| ∆Ct.vBMD (mg/cm^3^) | -1.3 | [-2.7 ; 3.0] | 0.3 | [-3.8 ; 2.6] |
| **Trabecular structure** | | | | |
| ∆Tb.N (mm^−1^) | -1.9 | [-3.6 ; 1.8] | -0.4 | [-5.5 ; 0.9] |
| ∆Tb.Th (mm) | -10.1 | [-16.2 ; -1.0] | -5.2 | [-7.9 ; 0.0] |
| ∆Tb.Sp (mm) | 2.7 | [0.6 ; 8.1] | 1.4 | [-0.2 ; 1.4] |

| Radial HR-pQCT | **Normal PTH** (n=9) | | **Persistent hyperPTH** (n=10) | |
| --- | --- | --- | --- | --- |
| **Density** | | | | |
| ∆Tb.vBMD (mg/cm^3^) | -13.0 | [-15.5 ; -4.1] | -6.5 | [-13.6 ; -1.0] |
| ∆Tt.vBMD (mg/cm^3^) | -6.6 | [-9.0 ; -4.5] | -2.6 | [-8.7 ; -1.0] |
| ∆Ct.vBMD (mg/cm^3^) | 0.34 | [-3.8 ; 2.6] | -2.3 | [-2.8 ; 3.9] |
| **Trabecular structure** | | | | |
| ∆Tb.N (mm^−1^) | 0.5 | [-5.1 ; 1.5] | -1.9 | [-3.6 ; 1.7] |
| ∆Tb.Th (mm) | -5.2 | [-16.2 ; -4.0] | -7.2 | [-16.6 ; -0.2] |
| ∆Tb.Sp (mm) | 1.5 | [0.0 ; 9.6] | 4.4 | [0.0 ; 7.7] |

| Radial HR-pQCT | **Pre-emptive KTx** (n=7) | | **Dialysis** (n=12) | |
| --- | --- | --- | --- | --- |
| **Density** | | | | |
| ∆Tb.vBMD (mg/cm^3^) | -6.5 | [-13.6 ; -0.5] | -9.0 | [-15.4 ; -4.5] |
| ∆Tt.vBMD (mg/cm^3^) | -3.6 | [-11.2 ; -1.9] | -6.4 | [-7.6 ; -1.1] |
| ∆Ct.vBMD (mg/cm^3^) | -2.4 | [-2.9 ; 2.6] | 0.3 | [-3.6 ; 3.0] |
| **Trabecular structure** | | | | |
| ∆Tb.N (mm^−1^) | -0.4 | [-4.0 ; 2.0 ] | -0.4 | [-5 ; 1] |
| ∆Tb.Th (mm) | -1.4 | [-1.0 ; 0.0] | -6.5 | [-16.0 ; -4.0] |
| ∆Tb.Sp (mm) | 2.0 | [-0.2 ; 8.0] | 3.5 | [0.0 ; 8.2] |

Results expressed as median [interquartile range, IQR] and % of the baseline value. vBMD: volumetric bone mineral density for total (Tt.vBMD), trabecular (Tb.vBMD), and cortical (Ct.vBMD) compartment; Tb.N: trabecular number; Tb.Th: thickness; Tb.Sp: separation; Ct.Ar: cortical area; Tt.Ar: total area; Ct.Th: cortical thickness; KTx: kidney transplantation

* p<0.025 when comparing HC and patients at KTx

# p<0.025 when comparing patients at KTx and 6 months post- KTx
